# Determination of 16 Selected Trace Elements in Children Plasma from China Economical Developed Rural Areas Using High Resolution Magnetic Sector Inductively Coupled Mass Spectrometry

**DOI:** 10.1155/2014/975820

**Published:** 2014-02-20

**Authors:** Xiaobing Liu, Jianhua Piao, Zhenwu Huang, Shuang-Qing Zhang, Weidong Li, Yuan Tian, Xiaoguang Yang

**Affiliations:** Key Laboratory of Trace Element Nutrition, Ministry of Health, Department of Trace Element Nutrition, National Institute of Nutrition and Food Safety, Chinese Center for Disease Control and Prevention, Beijing 100050, China

## Abstract

A rapid, accurate, and high performance method of high resolution sector field inductively coupled plasma mass spectrometry (HR-ICP-MS) combined with a small-size sample (0.1 mL) preparation was established. The method was validated and applied for the determination of 16 selected plasma trace elements (Fe, Cu, Zn, Rb, B, Al, Se, Sr, V, Cr, Mn, Co, As, Mo, Cd, and Pb). The linear working ranges were over three intervals, 0-1 **μ**g/L, 0–10 **μ**g/L and 0–100 **μ**g/L. Correlation coefficients (*R* ^2^) ranged from 0.9957 to 0.9999 and the limits of quantification (LOQ) ranged from 0.02 **μ**g/L (Rb) to 1.89 **μ**g/L (Se). The trueness (or recovery) spanned from 89.82% (Al) to 119.15% (Se) and precision expressed by the relative standard deviation (RSD %) for intra-day ranging from 1.1% (Zn) to 9.0% (Se), while ranged from 3.7% (Fe) to 12.7% (Al) for interday. A total of 440 plasma samples were collected from Chinese National Nutrition and Health Survey Project 2002 (CNNHS 2002), which represented the status of plasma trace elements for the children aged 3–12 years from China economical developed rural areas. The concentrations of 16 trace elements were summarized and compared by age groups and gender, which can be used as one of the basic components for the formulation of the baseline reference values of trace elements for the children in 2002.

## 1. Introduction

The importance of trace elements and their role in biological cycling and homeostasis has become evident in recent years [1, 2], due to deficiency of essential trace elements or excess of potentially toxic elements accumulation in human body for a long time, which are responsible for various diseases [3]. So the formulation of trace elements reference values is indispensable prerequisite for the evaluation of body health, as well as the cost-effectiveness analysis [4].

Plasma is one of the preferred biological fluids and widely used for biomonitoring purposes in the surveys involving trace elements. But it is often limited by a small-size sample, complicated matrix, simultaneous multielemental analysis, and a wide range of concentrations of analytes from trace (*μ*g/L) to ultratrace levels (ng/L-pg/L) [5]. Fortunately, high resolution sector field inductively coupled plasma mass spectrometry (HR-ICP-MS) is the most suitable equipment for the requirements because it exhibits an excellent sensitivity and precision, more superior ability to depress spectral interference, low volume sample consumption, and the rapid simultaneous analysis of multiple elements over a large range of concentrations [6]. Currently, the completed method of HR-ICP-MS combined with a simple-size biological sample preparation was established and applied for the multielemental analysis in practice, which can speed up analysis and limit potential sources of contamination.

So far, many studies involving trace elements across population have been carried out in different areas and countries, such as Italy [7, 8], Germany [9], UK [10], and Canada [11]. However, in China, there is no large-scale and multiregional survey including so many trace elements in population, especially in children, which is still scarce and fragmentary. However, children are usually faced with higher health risk of concern because of their special physiological susceptibility, behavior, lifestyles and dietary habits, and other related health factors [8]. In addition, many intervention programs involving trace elements on children had been conducted in recent years, but a partial cost-effectiveness analysis had not been performed for lacking of the original baseline values of trace elements. Therefore, the study to obtain the original baseline values of trace elements for children is really urgent and necessary, especially the different types of economic regions. Chinese National Nutrition and Health Survey Project 2002 (CNNHS 2002), which is a nationally representative and cross-sectional study, provides a good opportunity to examine the status of plasma trace elements of children in 2002.

The objectives of our study were to apply the optimized HR-ICP-MS combined with simple small-size sample preparation to determine 16 selected plasma trace elements (Fe, Cu, Zn, Rb, B, Al, Se, Sr, V, Cr, Mn, Co, As, Mo, Cd, and Pb) in children aged 3–12 years and to formulate the reference values of 16 selected trace elements, which could be considered as the original baseline levels for the representative children living in China economical developed rural areas in 2002.

## 2. Experimental

### 2.1. Materials and Reagents

High-pure water (18.2 Ω) was obtained from TKA Gen-Pure apparatus (Gen-Pure TKA, Niederelbert, Germany). The electronic grade nitric acid (HNO_3_) was used after additional purification by subboiling distillation in a PTFE still. Screw-capped polystyrene liners, 15 mL Corning Costar polypropylene centrifuge tubes were used for standard solution and sample preparation. All disposable pipette tips and plastic tubes were rigorously cleaned before use by immersion in 10% (v/v) HNO_3_ and rising with high-pure water.

Single-element standard solutions of 1000 mg/L of Fe, Cu, Zn, B, Al, Se, Mn, Mo, Co, Cr, Cd, Sr, Rb, V, Pb, and As were got from National Center for Standard Materials (Beijing, China). Internal standard elements, consisted of 1000 mg/L of Sc, Ga, Y, and Tl, were purchased from National Steel Materials Testing Center (Beijing, China). The certified reference materials used for internal quality control were Clichek Blood Plasma Control level 1 (Recipe, Munich, Germany).

### 2.2. Instruments Setting and Optimization

The HR-ICP-MS system was a Thermo Finnigan Element II model (Bremen, Germany) equipped with a 100 *μ*L/min concentric glass nebulizer, a water-cooled Scott double-pass spray chamber, and torch with guard electrode device, nickel interface cones. The major ICP-MS operational settings were listed: RF power, 1280–1300 W; Argon gas flow rates, 16.0 L/min (cool), 0.94 L/min (auxiliary), and 1.110–1.200 L/min (sample); sample uptake rate, 0.1-0.2 mL/min. Daily instrument optimization was performed by monitoring the sensitivity and stability for masses ^7^Li, ^115^In, and ^238^U in tune solution. The resolution (300, 4000, and 10000) for elements was selected by the interference that happened in plasma samples. The interday and intraday stability of equipment were kept constant as much as possible. The percentage of oxides and doubly charged ion formation (BaO^+^/Ba and Ba^2+^/Ba ratios) was maintained less than 0.003 and 0.03, respectively.

### 2.3. Preparation of Samples, Calibration Solutions, and Quality Control

The plasma samples were stored in deep frozen state at −80°C and kept very well without repeated freeze-thaw cycles prior to analysis. The selected plasma samples were reconstituted at room temperature, and then were shaken slightly and centrifuged at 2000 g for 15 minutes. 0.1 mL supernatant of plasma was diluted with 0.5% (v/v) HNO_3_ and internal standard solutions in 1 : 20 (v/v) up to 2 mL.

16 single-element standards (Fe, Cu, Zn, Rb, B, Al, Se, Sr, V, Cr, Mn, Co, As, Mo Cd, and Pb) were mixed in the calibration solutions and diluted with 0.5% (v/v) HNO_3_ and internal standard solutions and then prepared in correspondence to the concentrations of trace elements in human plasma.

The certified reference materials used for internal quality control were diluted according to the instructions of products, further prepared with 0.5% HNO_3_ (v/v). For those elements which are not certified in the commercially available samples, were performed by analyzing pooled plasma control samples fortified with appropriate aliquots (approximately 1-times baseline level) of trace elements.

### 2.4. Samples of Analysis

16 selected trace elements were determined by HR-ICP-MS using external standard quantification mode in class 1000 clean room. The determination of isotope was chosen according to the abundance, type of interference, and the amount of analytes expected in the matrix. For ^11^B, ^85^Rb, ^88^Sr, ^95^Mo, ^111^Cd, and ^208^Pb, the analyses were in low resolution (LR, 300 m/Δm), for ^27^Al, ^51^V, ^52^Cr,^55^Mn,^56^Fe, ^59^Co, ^63^Cu, ^66^Zn, the analyses were in medium resolution (MR, 4000 m/Δm), and, for ^75^As and ^77^Se, the analyses were in high resolution (HR, 10000 m/Δm). Internal standards (^45^Sc, ^69^Ga, ^89^Y, and ^205^Tl) were used and the final concentrations were 10 *μ*g/L, which may provide adequate means for dealing with nonspectral interference and instrumental drifts [12].

### 2.5. Source of Plasma Samples

The plasma samples were chosen from the list of blood species established in CNNHS 2002 using a random stratified sampling procedure with proportional allocation by gender and age groups. A total of 440 plasma samples were selected from the children (boys: 232, girls: 208) with an age of 8.02 ± 2.86 years living in China economical developed rural areas, which was equal to approximately 10% of the children blood species in that area.

### 2.6. Statistical Analysis

The distribution of 16 selected trace elements was not normal according to Kolmogorov-Smirnov's tests. Basic descriptive statistics were estimated from untransformed data including selected percentiles (P_5_, P_50_, and P_95_), geometric mean (GM), and the 95% confidence interval of the geometric mean (95% CI GM) of 16 selected trace elements for the children. Additionally, GM, 95% CI GM of 16 selected trace elements was described by gender in conjunction with age groups. The differences caused by gender and age groups were identified with the Mann-Whitney *U* test. A significant difference is *P* < 0.05. All statistical analyses were completed by using the statistical package SPSS Base 20.0 (SPSS, Chicago, IL, USA).

## 3. Results and Discussion

### 3.1. Analytical Characteristics

The in-house method validation parameters were obtained and listed in Table 1. The working linear ranges were over at least 5 calibration points, which were divided into three ranges such as 0–100 *μ*g/L, 0–10 *μ*g/L, and 0-1 *μ*g/L. The linear equations were established by over 20 replicates per calibration solution indicating linearity between signal and concentration, and correlation coefficient (*R* ^2^) ranged from 0.9957 (^27^Al) to 0.9999 (^63^Cu and ^56^Fe). The limits of quantitation (LOQ) ranged from 0.02 *μ*g/L (^85^Rb) to 1.89 *μ*g/L (^77^Se), calculated as 10 SD above the value for 0.5% HNO_3_ solution (*n* = 20) plus the dilution factor, which in this case was 20, considering the matrix effects and the excellent sensitivity of HR-ICP-MS.

Table 2 shows the trueness (or recovery) and precision of method, which were performed by checking against values given by the certified reference materials and by testing against pooled plasma samples fortified with a known quantity of reference standards. The resulting trueness (or recovery) spanned from 89.82% (Al) to 119.15% (Se). The precision was expressed by calculating the relative standard deviance (RSD% = (standard deviance/Mean) × 100) for intraday and interday, respectively. The RSD% for intraday indicated the stability of the determination in one day (one sample preparation, 10 replicate measurements of the same sample) and the RSD% for interday indicated the stability of the determination in 10 consecutive days (10 different sample preparations, 10 measurements in different days) [13]. The precision ranged from 1.1% (Zn) to 9.0% (Se) in RSD% for intraday and 3.7% (Fe) to 12.7% (Al) in RSD% for interday.

As shown above, the optimization method of HR-ICP-MS combined with the simple sample preparation with 0.5% HNO_3_ was applied for the determination of 16 selected trace elements from ng/L to *μ*g/L ranges. The 20-fold dilution causes the minor fluctuation at low concentrations for certain trace elements, but the method was reliable for the validation of methodological parameters and there was no coagulation or precipitation of plasma matrix components resulting in tubing or nebulizer blockages during 12 h of analysis. Compared with the other analytical procedures [12, 14], the method using 0.5% HNO_3_ directly diluted in 1 : 20 (v/v) is more rapid and free of contamination, especially in 0.1 mL volume of small-size sample consumption, low LOQ, and high sample throughput HR-ICP-MS, which can provide more flexibility in multielemental analysis.

### 3.2. Trace Elements Reference Values in Children Plasma

16 selected trace elements for 440 plasma samples are successfully determined and the detailed results are summarized in Table 3. The concentrations of Cd, Pb, V, Cr, and As are usually very low (ng/L), even below the LOQ. During the analysis, the concentration of Al in plasma sample is still easily affected because of its ubiquitous, so that the rigorous control of exogenous contamination is of great importance.

The further description of 16 selected plasma trace elements in children is presented by gender and age groups, which are listed in Tables 4 and 5.

Comparing between 3–6 years children and 7–12 years children, most of the GM concentrations of trace elements are higher for 3–6 years children with some exceptions (Rb, Sr, Cd, As, and Se) in boys and with some exceptions (Rb, Sr) in girls. Overall, the concentrations of Cr (*Z* = −2.622, *P* < 0.01), V (*Z* = −2.728, *P* < 0.01), Mn (*Z* = −2.470, *P* < 0.05), and Cu (*Z* = −6.071, *P* < 0.001) for 3–6 years children are significantly higher than 7–12 years children. The concentrations of V (*Z* = −2.478, *P* < 0.05) and Cu (*Z* = −3.900, *P* < 0.001) for 3–6 years children are significantly higher than 7–12 years children in boys, and the concentration of Cu is significantly higher in girls (*Z* = −4.479, *P* < 0.001).

In a comparison between boys and girls, the concentrations of B (*Z* = −2.908, *P* < 0.01), Sr (*Z* = −2.587, *P* < 0.01), and Fe (*Z* = −3.186, *P* < 0.001) for boys are significantly lower than girls, and the concentrations of B (*Z* = −2.371, *P* < 0.05), Sr (*Z* = −2.072, *P* < 0.05), and Fe (*Z* = −2.844, *P* < 0.01) are obviously lower in 7–12 years boys.

In general, in the children who lived in fixed areas for a long period of time without any major changes, the status of trace elements is representative and relatively stable. Age and gender are the major factors that affect the body burden of trace elements. Furthermore, the plasma trace elements usually associate with the age and gender after controlling for other potential confounders [15]. Age-related changes may be caused by behavior, dietary habits, and lifestyle. Gender-related changes might rely on a specific source of exposure in one sex or due to the difference in physiological factors or metabolic pathways. Comparing the status of trace elements in plasma of our study with other previous studies [14, 16, 17], most of trace elements such as B, Rb, Sr, Mo, V, Cr, Mn, Fe, Co, Cu, Zn, and Se are mostly similar to the wide ranges reported before and only the concentrations of Cd, Al, Pb, and As are at relatively low levels, which may be correlated with the surroundings. In addition, the limitation of this study is a relatively small sampling, which should be expanded in future studies.

## 4. Conclusion

In this study, we have established the method for the determination of 16 selected plasma trace elements in children. The method, combining HR-ICP-MS with the simply diluted 0.5% HNO_3_ by 1 : 20 (v/v), is an excellent method for rapid, free-contamination, small-size sample requirement and enough low LOQ for the most of plasma trace elements analysis. In addition, the concentrations of 16 selected plasma trace elements of the children from China economical developed rural areas were determined. The first representative data can be used as one of the basic components for the formulation of original baseline reference values of trace elements for the children aged 3–12 years in 2002, which can be helpful in the subsequent health evaluation and the cost-effectiveness analysis of health intervention programs.

## Figures and Tables

**Table 1:** 16 selected isotopes, linear working ranges and equations, correlation coefficient (*R* ^2^), and limits of quantitation (LOQ) considering the dilution factor (dilution factor = 20).

Isotope	Ranges (µg/L)	Linear equation (conc = *a* ∗ signal + *b*)	*R* ^2^	LOQ (*μ*g/L, *n* = 20)
^ 11^B	0–10	*y* = 1.425*e* ^−004^ *x* − 2.770*e* ^−003^	0.9997	0.38
^ 85^Rb	0–100	*y* = 9.255*e* ^−001^ *x* + 6.444*e* ^−002^	0.9998	0.02
^ 88^Sr	0–10	*y* = 1.113*e* ^−004^ *x* + 3.690*e* ^−002^	0.9996	0.06
^ 95^Mo	0-1	*y* = 1.871*e* ^−004^ *x* + 1.780*e* ^−004^	0.9989	0.03
^ 111^Cd	0-1	*y* = 1.449*e* ^−004^ *x* − 4.019*e* ^−005^	0.9979	0.03
^ 208^Pb	0-1	*y* = 1.056*e* ^−003^ *x* + 2.229*e* ^−003^	0.9989	0.17
^ 27^Al	0–2	*y* = 9.164*e* ^−004^ *x* + 1.492*e* ^−001^	0.9957	0.24
^ 51^V	0-1	*y* = 1.560*e* ^−003^ *x* + 1.059*e* ^−003^	0.9989	0.06
^ 52^Cr	0-1	*y* = 1.289*e* ^−003^ *x* − 6.643*e* ^−003^	0.9992	0.09
^ 55^Mn	0-1	*y* = 1.807*e* ^−003^ *x* − 4.863*e* ^−003^	0.9998	0.12
^ 56^Fe	0–100	*y* = 1.592*e* ^+000^ *x* + 6.966*e* ^−001^	0.9999	0.25
^ 59^Co	0-1	*y* = 1.586*e* ^−003^ *x* − 7.618*e* ^−004^	0.9995	0.06
^ 63^Cu	0–100	*y* = 9.054*e* ^−001^ *x* − 2.449*e* ^−001^	0.9999	0.48
^ 66^Zn	0–100	*y* = 2.325*e* ^−001^ *x* + 8.411*e* ^−001^	0.9996	0.45
^ 75^As	0-1	*y* = 2.041*e* ^−004^ *x* − 4.325*e* ^−004^	0.9969	0.56
^ 77^Se	0–10	*y* = 2.396*e* ^−005^ *x* − 9.134*e* ^−004^	0.9973	1.89

**Table 2:** Trueness (or recovery) and precision of the certified reference materials and the fortified pooled plasma analysis.

Element	Reference materials	Concentrations (µg/L)		RSD (%)		Trueness/recovery (%)
		Certified/spiked	Measured	Intraday (*n* = 10)	Interday (*n* = 10)	
Mo	Recipe level 1	1.82 ± 0.09	1.92 ± 0.12	3.2	8.8	105.49
Cd	Recipe level 1	2.37 ± 0.12	2.25 ± 0.10	4.1	9.3	94.73
V	Recipe level 1	1.45 ± 0.7	1.61 ± 0.03	4.7	10.9	111.22
Cr	Recipe level 1	3.56 ± 0.18	3.78 ± 0.12	3.8	5.6	106.25
Co	Recipe level 1	2.20 ± 0.11	2.31 ± 0.06	3.7	9.6	105.09
Cu	Recipe level 1	871.00 ± 43.55	835.01 ± 19.72	1.3	9.7	95.87
Zn	Recipe level 1	925.00 ± 46.25	873.12 ± 14.57	1.1	8.7	94.39
As	Recipe level 1	47.80 ± 2.40	52.75 ± 2.92	7.7	10.9	110.36
Se	Recipe level 1	80.00 ± 4	95.32 ± 6.59	9.0	11.2	119.15
B	Pooled	24.04	23.57	3.7	10.8	98.04
Rb	Pooled	244.3	254.21	2.8	9.6	104.06
Sr	Pooled	36.4	33.51	2.4	7.9	92.03
Pb	Pooled	0.42	0.39	4.8	8.6	92.85
Al	Pooled	3.83	3.54	7.4	12.7	89.82
Mn	Pooled	2.38	2.44	3.3	4.5	102.52
Fe^#^	Pooled	407.02	417.84	1.5	3.7	102.66

^#^Spike 0.5-times baseline.

**Table 3:** Concentrations of 16 selected plasma trace elements in children (*N* = 440) aged 3–12 years from China economical developed rural areas.

Element	*n* < LOQ	Percentiles (*μ*g/L)			GM (*μ*g/L)	95% CI GM (*μ*g/L)
		*P*_5_	*P*_50_	*P*_95_		
B	0	16.13	33.86	94.88	36.44	34.40–38.72
Rb	0	173.61	360.73	1281.50	405.80	384.50–428.62
Sr	0	30.33	58.40	95.99	56.63	54.71–58.85
Mo	0	1.48	2.30	3.97	2.36	2.28–2.42
Cd^a^	59	<LOQ	64.53	176.11	64.53^b^	59.86–68.42^b^
Pb^a^	9	217.0	576.14	1989.82	599.53	563.66–643.05
Al	47	<LOQ	1.40	8.87	1.40^b^	1.12–1.71^b^
V^a^	46	<LOQ	116.09	277.58	114.32	108.45–121.17
Cr^a^	6	137.70	408.22	6819.27	538.41	484.40–804.82
Mn	0	0.77	1.51	6.24	1.77	1.66–1.90
Fe	0	660.68	1304.13	2565.59	1375.51	1313.36–1442.48
Co^a^	56	64.73	195.94	452.46	181.94	171.64–193.73
Cu	0	673.26	974.01	1386.36	972.21	951.79–993.61
Zn	0	557.27	765.31	1027.16	754.74	741.47–770.39
As	68	<LOQ	1.17	7.14	1.51	1.37–1.68
Se	0	68.52	119.80	173.16	116.32	113.33–119.53

^a^ng/L, ^b^median and CI median.

**Table 4:** Concentrations (*μ*g/L) of 16 selected plasma trace elements in boys (*N* = 232) by age groups.

Element	GM	95% CI GM	3–6 years (*n* = 71)		7–12 years (*n* = 137)	
			GM	95% CI GM	GM	95% CI GM
B	33.4	31.30–35.99	35.51	31.70–40.53	32.31	29.56–35.55
Rb	395.89	368.26–425.68	362.34	324.79–400.17	415.14	375.44–462.18
Sr	54.46	51.98–57.16	54.01	50.31–57.91	54.71	51.45–58.31
Mo	2.40	2.31–2.50	2.48	2.33–2.63	2.36	2.25–2.48
Cd^ab^	64.53	59.46–70.70	58.88	52.19–70.46	67.67	60.71–73.95
Pb^a^	596.42	548.41–657.44	641.92	541.96–770.01	573.36	518.40–640.28
Al^b^	1.34	1.05–1.71	1.62	1.06–2.67	1.12	0.90–1.61
V^a^	112.51	103.75–122.17	132.53	117.09–152.93	103.05	92.87–113.43
Cr^a^	576.33	495.71–680.52	748.1	563.83–1047.27	501.07	419.89–602.85
Mn	1.72	1.56–1.88	1.89	1.63–2.22	1.63	1.45–1.83
Fe	1274.32	1191.60–1372.53	1371.04	1224.06–1546.84	1225.29	1131.34–1345.05
Co^a^	180.72	165.17–197.94	194.61	175.05–217.05	173.68	154.22–195.23
Cu	987.4	958.50–1016.89	1072.5	1029.30–1120.49	944.57	911.40–978.38
Zn	760.51	743.07–778.79	773.31	743.78–802.74	753.73	731.43–777.40
As	1.54	1.34–1.79	1.42	1.17–1.77	1.61	1.33–1.97
Se	118	113.91–122.26	117.15	110.06–125.08	118.45	113.50–123.57

^a^ng/L, ^b^median and CI median.

**Table 5:** Concentrations (*μ*g/L) of 16 selected plasma trace elements in girls (*N* = 208) by age groups.

Element	GM	95% CI GM	3–6 years (*n* = 71)		7–12years (*n* = 137)	
			GM	95% CI GM	GM	95% CI GM
B	40.17	36.81–43.96	42.52	36.98–48.67	39.0	35.29–43.66
Rb	417.16	383.77–453.30	390.64	333.78–459.67	431.6	390.49–481.66
Sr	59.15	56.10–62.61	58.4	53.31–64.03	59.53	55.65–63.55
Mo	2.30	2.20–2.40	2.41	2.22–2.61	2.25	2.15–2.36
Cd^ab^	64.21	57.25–70.45	65.51	59.05–77.20	61.06	54.40–67.51
Pb^a^	603.01	548.67–665.21	666.89	562.87–795.59	572.36	514.27–641.99
Al^b^	1.46	1.01–2.01	1.53	1.17–2.16	1.36	0.85–2.12
V^a^	116.37	107.83–125.76	128.86	115.05–143.77	110.38	99.51–122.55
Cr^a^	499.04	436.04–577.73	589.53	462.01–762.18	457.76	392.96–543.89
Mn	1.83	1.67–2.02	2.00	1.68–2.40	1.75	1.56–1.97
Fe	1497.88	1402.61–1611.11	1582.55	1399.60–1815.70	1455.8	1339.46–1585.82
Co^a^	183.31	170.64–196.81	193.74	171.97–220.78	178.13	161.80–195.06
Cu	955.54	926.00–985.77	1047.22	979.18–1112.75	911.23	883.09–939.70
Zn	748.36	728.40–769.65	773.29	739.76–813.93	735.75	713.39–759.50
As	1.47	1.28–1.70	1.51	1.16–1.97	1.45	1.24–1.69
Se	114.47	110.23–118.89	116.14	108.86–123.86	113.62	107.82–119.16

^a^ng/L, ^b^median and CI median.
